# High Responsivity Vacuum Nano-Photodiode Using Single-Crystal CsPbBr_3_ Micro-Sheet

**DOI:** 10.3390/nano12234205

**Published:** 2022-11-26

**Authors:** Xiangjun Zeng, Shasha Li, Zairan Liu, Yang Chen, Jun Chen, Shaozhi Deng, Fei Liu, Juncong She

**Affiliations:** State Key Laboratory of Optoelectronic Materials and Technologies, Guangdong Province Key Laboratory of Display Material and Technology, School of Electronics and Information Technology, Sun Yat-sen University, Guangzhou 510275, China

**Keywords:** photo assisted field emission, single-crystal CsPbBr_3_, high photo responsivity, nanoscale-channel-photodiode, photoconductive effect

## Abstract

Field electron emission vacuum photodiode is promising for converting free-space electromagnetic radiation into electronic signal within an ultrafast timescale due to the ballistic electron transport in its vacuum channel. However, the low photoelectric conversion efficiency still hinders the popularity of vacuum photodiode. Here, we report an on-chip integrated vacuum nano-photodiode constructed from a Si-tip anode and a single-crystal CsPbBr_3_ cathode with a nano-separation of ~30 nm. Benefiting from the nanoscale vacuum channel and the high surface work function of the CsPbBr_3_ (4.55 eV), the vacuum nano-photodiode exhibits a low driving voltage of 15 V with an ultra-low dark current (50 pA). The vacuum nano-photodiode demonstrates a high photo responsivity (1.75 AW^−1^@15 V) under the illumination of a 532-nm laser light. The estimated external quantum efficiency is up to 400%. The electrostatic field simulation indicates that the CsPbBr_3_ cathode can be totally depleted at an optimal thickness. The large built-in electric field in the depletion region facilitates the dissociation of photoexcited electron–hole pairs, leading to an enhanced photoelectric conversion efficiency. Moreover, the voltage drop in the vacuum channel increases due to the photoconductive effect, which is beneficial to the narrowing of the vacuum barrier for more efficient electron tunneling. This device shows great promise for the development of highly sensitive perovskite-based vacuum opto-electronics.

## 1. Introduction

Field electron emission possesses an intrinsically ultrafast response due to the near-instantaneous electron tunneling process and the scattering-free transport of electrons in its vacuum channel [1,2,3]. In recent years, the integration of field electron emission with other physical processes, such as laser excitation, for efficient electron emission and multi-functional devices has gained intense interests [4,5]. Field electron emission under continuous-wave laser exposure, which is termed photo-assisted field electron emission (PFE) [6,7], is one of the most popular strategies in this respect. The PFE process harvests photo-induced “hot” electrons for considerably enhanced electron emission. In comparison to the optical field electron emission (OFE), which is another type of laser-assisted process utilizing a high-power (~100 GWcm^−2^) femtosecond laser [8,9,10], the PFE process takes the advantage of moderate photo-excitation and high photoelectric conversion efficiency [11]. As a result, the demonstration of the PFE process in novel device structures can lead to high-performance vacuum photodiodes, which are promising for ultrafast photo detection, sensing, and information processing [2,12]. The PFE process in traditional vacuum electronic device structure is, however, limited by high driving voltage. In recent years, research advances in the scaling of vacuum channel length down to sub-50 nm have offered a competitive solution to address this challenge [13,14,15]. The driving voltage can be lowered to tens of volts in these sub-50 nm-channel devices, offering great opportunities for practical applications. So far, investigation on the PFE process in a sub-50 nm-channel device is still lacking. Moreover, improving the photo-responsivity of field emission materials is highly desirable. Plasmonic structures with strong electromagnetic field enhancement have been recently demonstrated to enhance the absorption of field emission materials [11,16,17,18,19]. For example, Qing Dai and co-workers proposed an optical antenna-coupled vacuum photodiode with nanogap forming at the cleaved edge of a metal-insulator-semiconductor structure, where a gold thin film with nano-hole array serves as the light-harvesting antenna cathode [12]. A significant increase in the external quantum efficiency (EQE) by two orders of magnitude to 4% was observed. In addition, a gold nanoparticle decorated few-layer graphene cathode working in plasmon-mediated electron emission mechanism was employed for photo-modulated field electron emission [11]. Gold nanoparticles act as plasmonic antennas to harvest light and provide hot electrons. The plasmon-generated hot electrons were injected from gold nanoparticles into the graphene and, thus, emitted into the vacuum. A field emission current density up to 155.6 mA∙cm^−2^ was obtained with a low-intensity laser (5 W∙cm^−2^) illumination. More interestingly, a PFE structure with the use of a p-type semiconductor, e.g., p-Si, as cathode has been reported to support a high photoresponsivity of 1.2 A∙W^−1^ and an EQE of 235% at a low bias of 5 V [20,21]. This result strongly suggests that p-type semiconductor, which is undervalued as a field emission material due to its limited electron supply, may offer new merit in the PFE process. However, the demonstration of high-performance PFE device structures with p-type cathode material is lacking. The underlying mechanism of photo-enhancement is still an open question.

Here, we report a well-designed field emission vacuum nano-photodiode, with a Si-tip anode and a single-crystal CsPbBr_3_ cathode separated by a nanoscale vacuum channel down to ~30 nm. Benefiting from the narrow anode-to-cathode separation and the ultra-high light absorption of the CsPbBr_3_, the photodiode exhibits a high photo responsivity (1.75 A∙W^−1^@15 V) with a low driving voltage (<20 V). The estimated EQE is up to 400% under the illumination of a 532-nm laser light. Furthermore, an ultra-low dark field emission current (50 pA @15 V) can be observed due to the high surface work function of the CsPbBr_3_ (~4.55 eV). This device shows great promise for developing highly sensitive perovskite-based vacuum opto-electronics.

## 2. Materials and Methods

### 2.1. Preparation of Single-Crystal CsPbBr_3_ Micro-Sheets

The CsPbBr_3_ micro-sheets were synthesized using an ambient-pressure chemical vapor deposition method [22]. Fresh-clave muscovite mica chips were used as the substrate. CsBr and PbBr_2_ powders with a molar ratio of 1:1 were separately placed on either side of a corundum boat. The boat was then placed in the heating zone of the tube-furnace. Air flow (100 standard cubic centimeters per minute) was introduced into the tube-chamber from the PbBr_2_ side. The tube-chamber was maintained at atmospheric pressure during the growth. A three-step heat-treatment process was performed for the growth of CsPbBr_3_. Firstly, the temperature was increased from room temperature to 400 °C in 30 min and maintained for 20 min. Secondly, the temperature was further increased to 650 °C in 15 min and slowly cooled down to 640 °C in 20 min. Finally, the tube-furnace was naturally cooled to room temperature.

### 2.2. Device Fabrication and Characterizations

In the micro-fabrication of the field emission vacuum nano-photodiode, the SiO_2_ films were deposited using plasma-enhanced chemical vapor deposition (PECVD, Plasmalab 80 Plus, OXFORD INSTRUMENTS, Oxford, UK) and the metal films were deposited using magnetron sputtering. Both the electron beam lithography (e-Line 150, Raith, Dortmund, Germany) and the UV-photo lithography (URE_2000S, IOE, Beijing, China) were employed for pattern defining. The dry etching of SiO_2_ and Si was performed using inductively-coupled-plasma etching (ICP, Plasmalab 100, OXFORD INSTRUMENTS, Oxford, UK). A scanning electron microscope (SEM, SUPRA 55, Zeiss, Oberkochen, Germany) and an optical microscope (BX43, OLYMPUS, Tokyo, Japan) were employed for morphology inspections. The crystalline structure of the CsPbBr_3_ micro-sheets were identified using a high-resolution transmission electron microscope (HRTEM, Titan3 G2 60-300, FEI, Hillsboro, OR, USA). A photoluminescence (PL) spectroscope (Invia Reflex, Renishaw, Gloucestershire, UK), an X-ray diffractometer (XRD, D-MAX 2200 VPC, Rigaku, Tokyo, Japan), an atomic force microscope (AFM, NTEGRA Spectra, NT-MDT, Moscow, Russia), and a Kelvin probe force microscopy (KPFM, Dimension fastscan, Bruker, Massachusetts, USA) were used to obtain the photoluminescence spectra, the crystalline structure, the surface topography, and the surface work function, respectively. Field electron emission measurements were performed in a high-vacuum (~10^−8^ Torr) chamber at room temperature. Before the field electron emission tests, the vacuum chamber and the samples were baked at 300 °C for 30 h for degassing. A picoammeter (6487, Keithley, Washington DC, USA) was used for power supply and current monitoring. The photo-response tests of the vacuum nano-photodiodes were performed under the illumination of focused super-continuum white-light (SC-Pro 400–2400 nm, OYSL Photonics, Wuhan, China) with long- and short-wave-pass filters (BLP01/FF01/BSP01, Semrock, New York, USA), and a 532 nm laser (LE-LS-532-100TA, LEOPTICS, Shenzhen, China), respectively. The light spot area was 0.04 cm^2^, which could fully cover the CsPbBr_3_ vacuum nano-photodiode, while the laser was irradiated to the upper surface of the CsPbBr_3_ cathode.

### 2.3. Finite Element Simulations

A numerical simulation was performed using finite element methods. We built a 2-dimensional axis symmetric model. The geometrical parameters were set according to the fabricated vacuum nano-photodiode. The Si-tip with a tip curvature radius of 5 nm is 1 μm in height. The thickness of the metal layer and the SiO_2_ layer is 200 nm and 830 nm, respectively. The separation between the CsPbBr_3_ and the Si-tip is 30 nm. The metal gate aperture was set as 3.5 μm. The size of the CsPbBr_3_ was set as 8 μm × 8 μm, with a thickness varying from 500 nm to 2.5 μm. The CsPbBr_3_ was taken as being lightly p-type doped with a hole concentration of 10^13^ cm^−3^. The Si-tip was taken as being heavily n-type doped with an electron concentration of 10^19^ cm^−3^. The CsPbBr_3_ was grounded, while the Si-tip was biased to a positive voltage of 15 V in the simulation. The simulation model would address two coupling processes. Firstly, the electric field induced by the anode voltage would cause electron accumulation on the surface of the CsPbBr_3_. Secondly, the redistribution of charges in the CsPbBr_3_ cathode would cause the change in the electric field in the vacuum. The two coupling processes would finally reach a balance-state, which would be determined by the potential continuity boundary condition at the interface between the CsPbBr_3_ and the vacuum region.

## 3. Results and Discussion

### 3.1. Single-Crystal CsPbBr_3_ Micro-Sheets

**Figure 1:**
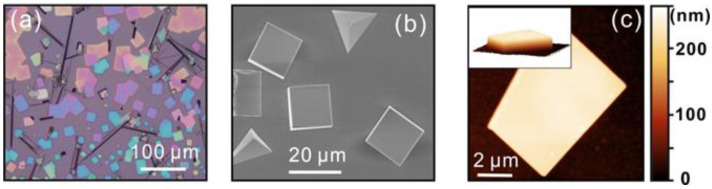
The morphology of CsPbBr_3_ micro-sheets. (**a**,**b**) are the typical optical microscopy and SEM images of the CsPbBr_3_ micro-sheets, respectively. (**c**) is the AFM surface morphology of a CsPbBr_3_ micro-sheet with the 3D morphology in the inset.

**Figure 2:**
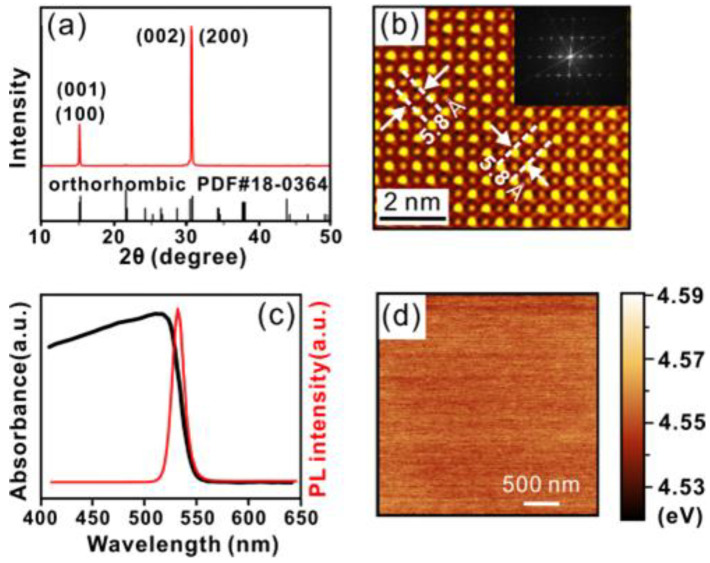
(**a**) The typical XRD spectrum (in red) of the as-grown CsPbBr_3_ micro-sheets, with an orthorhombic phase of CsPbBr_3_ crystal (PDF #18-0364) for comparison (black lines). (**b**) The typical HRTEM and the corresponding fast Fourier transform (FFT) pattern (the inset) of the CsPbBr_3_ micro-sheet. The white arrows indicate a lattice space of 0.58 nm. (**c**) The UV-vis absorption spectrum (in black) and the PL spectrum (in red) of the as-grown CsPbBr_3_ micro-sheets. (**d**) The typical KPFM image of a CsPbBr_3_ micro-sheet showing the surface work function of the CsPbBr_3_.

Figure 1a shows the typical optical microscopy image of the as-prepared CsPbBr_3_ sheets (in square shape) on the mica substrate. The color variation in Figure 1a results from the light interference of the micro-sheets with different thickness. The area of the square CsPbBr_3_ sheets is typically 20 × 20 μm^2^ (Figure 1b). Most of the micro-sheets are 200–2000 nm in thickness. Figure 1c is the typical AFM surface topography of a CsPbBr_3_ micro-sheet with a thickness of ~203 nm, showing a smooth surface with a root-mean-square roughness of 0.3 nm. The characterization of the crystallographic properties of the micro-sheets were performed using XRD. The peaks (Figure 2a) can be indexed to the orthorhombic phase of the CsPbBr_3_ (PDF #18-0364). Strong peaks from (001), (100), (002), and (200) planes from the CsPbBr_3_ crystal were observed. The strong diffraction peak with a narrow full width at half maximum (FWHM) at 13° indicates that the CsPbBr_3_ grows along the (001) and (100) orientation. The HRTEM image in Figure 2b shows a lattice spacing of 0.58 nm, confirming the (100) and (001) growth direction for the high-quality single-crystal CsPbBr_3_ micro-sheets. The UV-vis absorption spectrum and the room temperature PL spectrum of the CsPbBr_3_ sheet are shown in Figure 2c. It indicates that the cut-off absorption wavelength is ~543 nm, suggesting a bandgap of ~2.25 eV. The PL spectrum of CsPbBr_3_ exhibits a sharp peak with a FWHM of ~14.5 nm, which indicates a high quantum yield of the material. The FWHM of the PL peak is narrower than those of most II-VI group compound semiconductor nanostructures and even narrower than those of CsPbBr_3_ quantum dots or nanoparticles [23,24]. The excitonic emissions of the as-grown microcrystals result from the recombination of the delocalized and strongly interacting Wannier–Mott excitons, suggesting that the CsPbBr_3_ micro-sheets are free from defects and surface disorders [25,26,27,28]. The typical KPFM image of a CsPbBr_3_ micro-sheet is shown in Figure 2d. The average surface work function of the CsPbBr_3_ is 4.55 eV.

### 3.2. CsPbBr_3_ Vacuum Nano-Photodiode and the Photo-Responsivity

**Figure 3:**
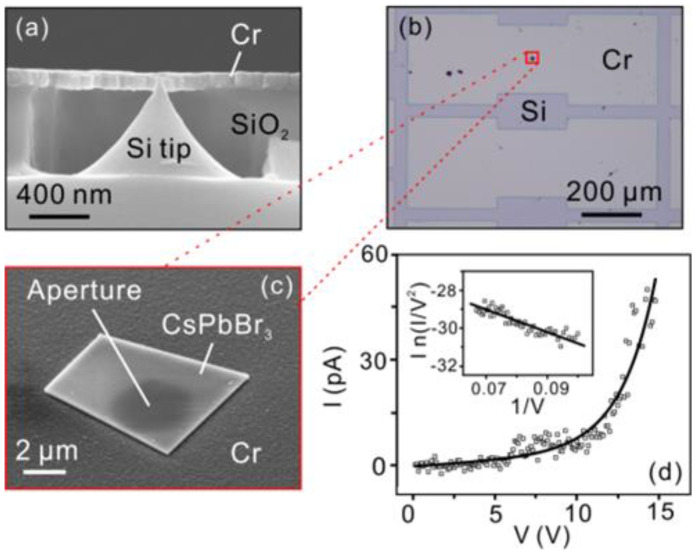
Fabrication of the CsPbBr_3_ vacuum nano-photodiode and its field emission characteristics. (**a**) The cross-sectional SEM image of the gated Si-tip. (**b**) The optical microscopy image showing the full view of the vacuum nano-photodiode. (**c**) The SEM image (45°-top-view) showing a vacuum nano-photodiode consisted of a CsPbBr_3_ micro-sheet covering the gate aperture. (**d**) The typical field electron emission I–V curve and the corresponding F–N plot (the inset) of the CsPbBr_3_ vacuum nano-photodiode.

The CsPbBr_3_ vacuum nano-photodiode was constructed using a gated Si nano-tip (Si-tip with a surrounding Cr electrode) and a CsPbBr_3_ micro-sheet. The gated Si-tip was fabricated following a well-developed top–down procedure that can be found in references [29,30]. Briefly, Si tips were fabricated using isotropic plasma etching and sharpened by thermal oxidation. Thin films of SiO_2_ and chromium were deposited on the tips in sequence and a self-aligned process was performed to locally remove the Cr and SiO_2_, forming a gated Si-tip (Figure 3a). The CsPbBr_3_ sheet was transferred onto the gated Si-tip using a dry transfer method. The CsPbBr_3_ micro-sheets were exfoliated from the mica via a polydimethylsiloxane (PDMS) film. The selected CsPbBr_3_ was aligned to the gate aperture (3.5 μm in diameter) under an optical microscope and placed onto the Cr layer to cover the gate aperture (Figure 3b,c). An integrated vacuum nano-photodiode using the CsPbBr_3_ as the cathode and the Si-tip as the anode was constructed. The separation between the CsPbBr_3_ cathode and the Si-tip anode was determined using the altitude difference between the tip apex and the Cr surface, which could be controlled by adjusting the thickness of the SiO_2_ layer. Typically, the cathode–anode separation of the vacuum nano-photodiode is ~30 nm. Figure 3d shows the typical field emission current–voltage (I–V) and Fowler–Nordheim (F–N) curves of the CsPbBr_3_ vacuum nano-photodiode measured in the dark, respectively. During the field emission measurements, the CsPbBr_3_ micro-sheet was grounded, while the Si-tip was biased to a positive voltage. Typically, the emission current increased exponentially with the applied voltage. The corresponding F–N plot fits onto a straight line, suggesting that the electron emission arises from the FN tunneling. An emission current of 50 pA was obtained at a voltage of 15 V, corresponding to a local electric field of 5 × 10^3^ MV∙m^−1^ at a 30-nm anode–cathode separation. The emission area of the CsPbBr_3_ was estimated to be ~100 nm^2^ according to the apex diameter of the Si-tip anode. Our results demonstrate that the CsPbBr_3_ vacuum nano-photodiode integrated with a nanoscale vacuum channel provides the merits of low driving voltage and ultralow dark field emission current. The ultralow dark field emission current in our structure is attributed to the nanoscale emission area and the relatively high surface work function of the CsPbBr_3_ (Figure 2d).

**Figure 4:**
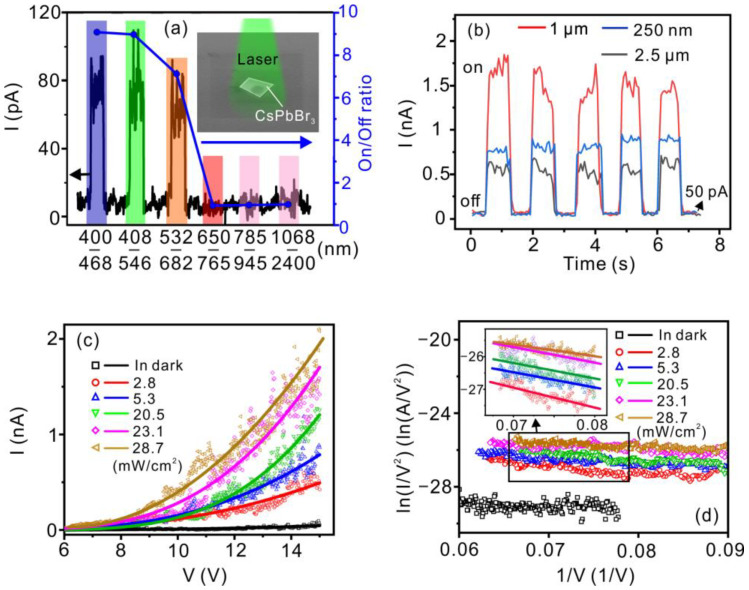
The photo-response characteristics of the CsPbBr_3_ vacuum nano-photodiode. (**a**) The photo response (dark line) and on/off ratio (blue line) of the CsPbBr_3_ vacuum nano-photodiode at 10 V under the illumination of light with different wavelengths at a power density of 3.1 × 10^−4^ mW∙m^−2^. The schematic showing the CsPbBr_3_ vacuum nano-photodiode under the light illumination is shown in the inset. (**b**) The current–time (I–t) curves of the nano-photodiodes obtained by switching a 532-nm laser light. The laser power was fixed at 23.1 mW∙cm^−2^. The Si-tip anode was biased at 15 V. The nano-photodiodes were constructed with CsPbBr_3_ micro-sheets in thickness of 250 nm, 1 μm, and 2.5 μm, respectively. (**c**,**d**) are the field electron emission I–V curves and the corresponding F–N plots of the vacuum nano-photodiode under the 532-nm light illumination with an incident power density from 0–28.7 mW∙cm^−2^. The used CsPbBr_3_ has a thickness of 1 μm.

Figure 4a shows the photo-response (black curve) and on/off ratio (blue line) of the vacuum nano-photodiode. The field electron emission of the CsPbBr_3_ vacuum nano-photodiode is sensitive to light illumination in the wavelength of 400–682 nm. An on/off ratio up to 9 was obtained in the spectral range of 400–545 nm, while no photo-response was observed in the range of 650–2400 nm. Our findings strongly suggest that the photo-response is related to the band-to-band absorption edge (543 nm) of the CsPbBr_3_ (Figure 2c). The influence of the thickness of CsPbBr_3_ on the photo-response characteristics was further investigated under a 532-nm laser illumination with a power density of 23.1 mW∙cm^−2^. The current–time (I–t) curves of the CsPbBr_3_ vacuum nano-photodiodes with three sets of thickness (250 nm, 1 μm, and 2.5 μm) are shown in Figure 4b. The photo-induced field emission current obtained from the vacuum nano-photodiodes has an optimal value when the thickness of the CsPbBr_3_ layer is 1 μm. The power dependence measurements on such device structure show that the field emission current can be effectively modulated by the incident power (Figure 4c). Typically, an emission current of 500 pA was obtained at 15 V with a power density of 2.8 mW∙cm^−2^, which was ten times that in the dark state (i.e., 50 pA@15 V). An emission current up to 2.1 × 10^3^ pA was obtained at the same bias with a power density of 28.7 mW∙cm^−2^, corresponding to an on/off ratio of 42. All the FN plots fit well onto a straight line (Figure 4d), suggesting that the electron emission arises from the FN tunneling rather than the thermal-assisted emission under light illumination. According to the FN theory [31], the slope of the FN plot (*K_FN_*) is negatively related to the effective work function (*ϕ_eff_*) of the cathode:

(1) $$K_{\mathrm{FN}}=-\frac{A\phi_{\mathrm{eff}}{}^{3/2}}{\beta }$$

In this formula, the constant A is 6.83 V∙eV^−3/2^∙nm^−1^ and β is the geometrical field enhancement factor. It is reasonable to take β as a constant since no morphology change was observed in the measurements. As indicated in the inset of Figure 4d, the FN plots show up-bending features with the increase in laser power. The corresponding *K_FN_* increases from −35 to −13, with the light power density increases from 0 to 28.7 mW∙cm^−2^. The calculated *ϕ_eff_* of the CsPbBr_3_ cathode decreases from 4.55 to 2.35 eV, with a reduction of 48.4%. The obvious change in *ϕ_eff_* is attributed to the generation of photo-induced hot electrons in the CsPbBr_3_ that raise its Fermi energy level. Furthermore, the vacuum nano-photodiode is sensitive to the illumination light in the wavelength of 400–682 nm in relatively low power density (2 mW∙cm^−2^), which indicates that the photo-response of the vacuum nano-photodiode originates from photo field emission rather than optical field emission in the CsPbBr_3_ cathode.

Key figure-of-merit parameters of the vacuum nano-photodiode, such as photoresponsivity (R = I_PH_/P_light_) [32] and specific detectivity (D* = R/(2qI_d_)^1/2^), were calculated to examine the performance of the CsPbBr_3_ vacuum nano-photodiode. Here, I_PH_ is the difference between the photocurrent and the dark current measured at the same applied voltage. P_light_ is the effective light power. q is the elementary charge, and I_d_ is the dark current. A photoresponsivity up to 1.75 A∙W^−1^ was obtained at 15 V with an illumination light power of 5.3 mW∙cm^−2^. The corresponding D* value was about 1.4 × 10^11^ Jones (Jones = cm∙Hz^1/2^·W^−1^). An EQE up to 400% was calculated using the following formula: EQE = Rhc/λq, where h is the Planck constant, c is the light velocity, and λ is the wavelength. Table 1 lists the typical performance parameters of the vacuum nano-photodiodes from previously reported studies and this work. It can be seen that our CsPbBr_3_ vacuum nano-photodiode presents the lowest dark current, which is several orders lower than those obtained from the other reported vacuum photodiodes due to the small emission area and relatively high work function of the CsPbBr_3_ cathode. More importantly, the CsPbBr_3_ vacuum nano-photodiode also exhibits the highest photoresponsivity and EQE with a relatively low operating voltage, showing its great potential for constructing highly sensitive integrated field emission vacuum photodiode.

**Figure 5:**
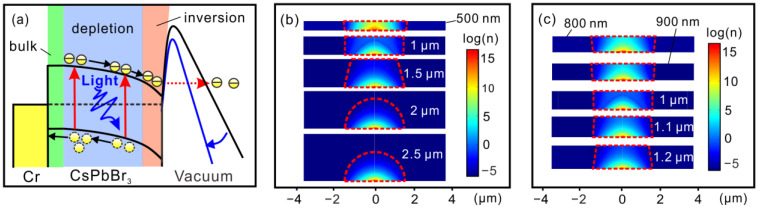
The underlying mechanism for the photo-response characteristics of the CsPbBr_3_ vacuum nano-photodiode. (**a**) The energy band diagram showing the electron emission process of photo-generated carriers in the CsPbBr_3_ vacuum nano-photodiode illuminated by a 532-nm laser light. (**b**) The cross-sectional view of the electron distributions in the CsPbBr_3_ cathodes with a thickness varying from 500 nm to 2.5 μm. (**c**) The cross-sectional view of the electron distributions in the CsPbBr_3_ cathodes with a thickness varying from 800 nm to 1.2 μm. The electron distributions showing in (**b**,**c**) were obtained at a bias of 15 V in the numerical simulation. Red dashed boxes mark the location of the depletion region in the CsPbBr_3_ cathode.

The ultra-high photoresponsivity (1.75 A∙W^−1^@15 V) and remarkable EQE (400% at 532 nm) of our vacuum nano-photodiode indicate that an efficient photo-current can be induced in a small emission area. The underlying mechanism for the outstanding photo-response characteristics is discussed further in Figure 5a,b. The influence of the Cr/CsPbBr_3_ heterojunction on the photo-response characteristics of our structure was excluded at the beginning due to the small work function difference (0.05 eV) between Cr and CsPbBr_3_. The work function of Cr is 4.5 eV [34], while that of CsPbBr_3_ is 4.55 eV (Figure 2d). It is well-known that a depletion region can be generated in p-type cathode as a result of electron accumulation during the field electron emission process. The built-in electric field in the CsPbBr_3_ depletion region can effectively separate and extract the photogenerated carriers. We, therefore, consider the influence of the depletion region first. A finite element based on numerical simulations were performed to investigate the charge distribution in the CsPbBr_3_ cathode in the dark state. In the simulations, the CsPbBr_3_ micro-sheets with a thickness varying from 500 nm to 2.5 μm were taken as being lightly p-type doped with a hole concentration of 10^13^ cm^−3^ [35]. The geometrical parameters were set according to the SEM characterization presented in Figure 3c. The Si-tip was biased at 15 V. Figure 5b presents the charge distribution in the CsPbBr_3_ cathode with different thicknesses. It can be seen that electrons are attracted by the local electric field and accumulated in the central region of the CsPbBr_3_ cathode, leading to an inversion layer with a high electron concentration of ~10^15^ cm^−3^. A carrier depletion region is accordingly formed. As illustrated in Figure 5b, the central region of the CsPbBr_3_ cathode is totally depleted when its thickness is no more than 1 μm. The location of the depletion region in the CsPbBr_3_ cathode is moving away from the upper surface of the CsPbBr_3_ cathode gradually when the cathode thickness increases from 1.5 μm to 2.5 μm. A hemispherical depletion region is formed when the CsPbBr_3_ cathode is thicker than 2 μm, corresponding to a depletion radius of 1.7 μm. It is worth noting that the incident light is largely absorbed in the upper surface of the CsPbBr_3_ cathode due to its superior absorption characteristics. The absorption rate has been reported to decrease exponentially with increasing distance from the incident surface [36]. The depletion region buried in the deep surface of the thick CsPbBr_3_ cathode will lead to an inefficient electron–hole separation due to the spatial mismatch between the region with strong built-in electric field and the region with sufficient electron–hole generation. It is also important that the CsPbBr_3_ cathode shows an optimal thickness. For example, the photocurrent of the 250-nm CsPbBr_3_ cathode is lower than that of the 1-μm CsPbBr_3_ cathode, although both of them are totally depleted. It is because the light absorption rate is closely related to the thickness of the cathode. The light absorption rate is written as η = 1 − e^-αz^, where α is the light absorption coefficient, i.e., 5 × 10^4^ cm^−1^ at 532 nm for the CsPbBr_3_ [37], and z is the thickness of the material. The light absorption rates of the CsPbBr_3_ are ~71% and ~99% when the thicknesses are 250 nm and 1 μm, respectively. The electron distributions of the CsPbBr_3_ cathodes with a thickness of around 1 μm were further investigated (Figure 5c). The results indicate that the optimal photo-response, with both large depletion volume and high absorption rate, could be obtained at 1 μm. The simulation results demonstrate the importance of the depletion region in facilitating the photo-induced field electron emission. It can also explain the thickness dependence of photo-response of the CsPbBr_3_ cathode observed in our experiments (Figure 4b).

In addition to the efficient electron–hole separation in the depletion region, the photoconductive effect may also contribute to the enhanced optoelectronic properties of the vacuum nano-photodiode. In our structure, the electron emission is mainly limited by the large vacuum barrier rather than the electron supply [38], which can be indicated by the linear feature of the F–N curves (Figure 3d). The large photoconductivity of the CsPbBr_3_ cathode can help increase the voltage drop on the vacuum barrier, leading to an exponential increase in the field emission current due to the narrowing of the barrier [39]. We take the high external field to be accountable for the high EQE of 400% in the CsPbBr_3_ vacuum nano-photodiode. The influence of a high electric field can be twofold. On the one hand, the photogenerated carriers are well-separated from the electron–hole pairs, with the photogenerated electrons accumulating in the central region of the CsPbBr_3_ cathode (Figure 5b). The recombination of photogenerated carriers is suppressed. On the other hand, electrons are extracted into the vacuum from the CsPbBr_3_ cathode due to the presence of the high electric field. Correspondingly, the electrons will be injected from the Cr electrodes to the CsPbBr_3_ cathode in order to maintain charge neutrality. The injection occurs until the photogenerated carriers recombine radiatively or non-radiatively in the device interior. Therefore, a single absorbed photon in our structure could generate much more conductible electrons, resulting in an over 100% EQE value.

## 4. Conclusions

We demonstrated a highly photosensitive CsPbBr_3_-based vacuum nano-photodiode operating at a low voltage of 15 V. This nano-photodiode operates in a visible region (400–680 nm), exhibiting high responsivity (R = 1.75 A∙W^−1^), detectivity (D* = 1.41 × 10^11^ Jones), photoconductive on/off ratio (42), and external quantum efficiency (EQE = 400%). Meanwhile, the device also shows an ultra-low dark current (50 pA), suggesting a low power consumption in the off-state. The enhanced photoelectric performance of the vacuum nano-photodiode is attributed to the efficient electron–hole separation in the CsPbBr_3_ depletion region and to the vacuum barrier narrowing due to the photoconductive effect. As a field emission cathode, single-crystal CsPbBr_3_ micro-sheets are promising for the applications in field emission displays and other vacuum nanodevices. It also provokes further research on the fast photo-response in the integrated CsPbBr_3_-based vacuum nano-photodiode, which has application prospects in the field of photo-modulated electron emission, ultrafast visible light detection, and photo-controlled triode for ultrafast light information processing.

## Figures and Tables

**Table 1:** Comparison of typical vacuum photodiode performance parameters.

Structure	Cathode Material	Photoresponsivity	EQE	Dark Current	Operating Voltage
Antenna-coupled nano diode [12]	Au	0.6 mA∙W^−1^	4%	300 pA	1–2 V
Si-Graphene [21]	P-doped Silicon	1.2 A∙W^−1^	235%	~100 nA	5 V
Black silicon cathode [5]	P-doped Silicon	1.85 mA∙W^−1^	0.01%	250 nA	>1000 V
Diamond film-Cu [33]	Diamond	~10 mA∙W^−1^	\	\	90–150 V
CsPbBr_3_-Si tip (This work)	Single-crystal CsPbBr_3_	1.75 A∙W^−1^	400%	50 pA	15 V

## Data Availability

The data presented in this study are available on request from the corresponding author.
